# Obese Children, Adults and Senior Citizens in the Eyes of the General Public: Results of a Representative Study on Stigma and Causation of Obesity

**DOI:** 10.1371/journal.pone.0046924

**Published:** 2012-10-12

**Authors:** Claudia Sikorski, Melanie Luppa, Elmar Brähler, Hans-Helmut König, Steffi G. Riedel-Heller

**Affiliations:** 1 Integrated Research and Treatment Center (IFB) Adiposity Diseases, University of Leipzig, Leipzig, Germany; 2 Institute of Social Medicine, Occupational Health and Public Health, University of Leipzig, Leipzig, Germany; 3 Department of Medical Psychology and Medical Sociology, University of Leipzig, Leipzig, Germany; 4 Department of Medical Sociology and Health Economics, Hamburg-Eppendorf University Medical Center, Hamburg, Germany; Chiba University Graduate School of Medicine, Japan

## Abstract

Obese individuals are blamed for their excess weight based on causal attribution to the individual. It is unclear whether obese individuals of different age groups and gender are faced with the same amount of stigmatization. This information is important in order to identify groups of individuals at risk for higher stigmatization and discrimination. A telephone interview was conducted in a representative sample of 3,003 participants. Experimental manipulation was realized by vignettes describing obese and normal-weight children, adults and senior citizens. Stigmatizing attitudes were measured by semantic differential. Causal attribution was assessed. Internal factors were rated with highest agreement rates as a cause for the vignette's obesity. Lack of activity behavior and eating too much are the most supported causes. Importance of causes differed for the different vignettes. For the child, external causes were considered more important. The overweight vignette was rated consistently more negatively. Higher educational attainment and personal obesity were associated with lower stigmatizing attitudes. The vignette of the obese child was rated more negatively compared to that of an adult or senior citizen. Obesity is seen as a controllable condition, but for children external factors are seen as well. Despite this finding, they are faced with higher stigmatizing attitudes in the general public, contradicting attribution theory assumptions. Internal and external attribution were found to be inter-correlated. Obese children are the population most at risk for being confronted with stigmatization, making them a target point in stigma-reduction campaigns.

## Introduction

About 20% of the adult population in Western countries is obese, and prevalence rates are rising [1]. Obesity is one of the major health problems in the developed world. It is associated with severe consequences for the individual in terms of higher mortality and worse health outcomes. Apart from bio-medically defined pathways to negative health outcomes, psychosocial consequences of being overweight and obesity and their interaction with biomedical pathways receive increasingly scientific attention.

Obese individuals are commonly blamed for their excess weight, and negative stereotypes such as lack of self-discipline are pervasive [2]. Connecting individuals to negative stereotypes leads to social distance toward these individuals and to discrimination. Discrimination, in turn, feeds back negative stereotyping, re-cycling the stigmatization process [3]. Stigmatizing attitudes towards obese individuals emerge in the context of beliefs about controllability and responsibility for the excess body weight [4]; [5]. According to attribution theory, disease stigma evolves when a disease is seen to be within the individual's control, hence if it is connected rather with internal (behavior and individual) than external (environment) and genetic factors [6].

Puhl and Heuer (2010) outlined a wide range of negative consequences of stigmatization in terms of individual and public health matters. On an individual level, perceived weight stigma might worsen unhealthy eating and activity behavior, induce or enhance psychological problems, and lead to inadequate help-seeking behavior and decreased health care utilization for obesity-related health problems [7]. It therefore directly interacts with biomedical pathways of obesity and impaired health outcomes. Ignoring weight stigma in public health campaigning will lead to impaired prevention efforts and a deepening social inequality, further exacerbating the obesity situation.

So far, research on weight stigma has mainly been based on selected samples in special settings, e.g. students [8]. A systematic review on population-based studies on weight stigma revealed only 5 studies conducted in 2 countries [9]. Comparability was hindered by the utilization of heterogeneous instruments. Especially stigmatizing attitudes were only reported in one study. In this German study, prevalence of definite stigmatizing attitudes was about 20% [10]. The influence of social desirability, however, can be expected to be substantial, since the study group used a direct questionnaire. Studies with more indirect ways to measure negative attribution are lacking. Causal attribution was only partly addressed within the reviewed studies. Individual causes were agreed on most often, while genetics seem to play only a minor role in the public's eye [11]–[13]. Although weight stigma in general was found to be common [14]; [15], so far, nothing is known whether obese individuals of different age groups (children, adults, senior citizens) and gender are faced with the same amount of stigmatization. There are only few studies that investigate weight prejudice in mothers toward obese children and adults. The authors found weight bias in children to be less prevalent than towards obese adults [16]. This information is important in order to identify groups of individuals at risk for higher stigmatization and discrimination.

This study therefore aims at (a) investigating the prevalence and answering patterns of stigmatizing attitudes in the German general public and (b) determining the lay public's view on causal attribution of obesity. Furthermore, by depicting six different vignettes, (c) effects of age and gender of obese individuals as well as other determinants of stigmatizing attitudes are investigated.

## Methods

### Study Design

The survey was conducted as a computer assisted telephone interview (CATI) in German language by USUMA, a leading market, opinion and social research institute in Germany which has conducted several population-based surveys in stigma research [17]; [18]. Data was collected from February to April 2011. Sampling was based on random digital dialing, drawing from the Association of German Market and Social Research Agency's (ADM) sample base that includes registered and non-registered telephone numbers. To ensure the sample is representative of the German population, all regions in Germany were included in the sampling process. Within a randomly selected household, the Kish-Selection-Grid was applied when randomly selecting the person in the household (at least 18 years of age) to be interviewed [19]. This ensured equal probability of participation for each member of the household. All interviewers were trained to conduct interviews by members of this study team. At the beginning of the interview, respondents were told that this was a survey on the health and living environment of people in Germany. Weight prejudice was not mentioned to avoid participation bias (table 1). All questions regarding weight were introduced as being necessary to optimize the following sets of questions.

**Table 1:** Socio-demographic characteristics of the samples compared to the German general population.

	Total Sample (n = 3003)	Reduced sample I (n = 2459)	German Population 12/2009^1^
Women	52.8	51.4	51.0
Age group			
<20	4.9	3.8	18.8
21–40	22.4	22.4	24.3
41–60	37.2	38.5	31.0
60–80	31.5	31.9	20.8
>81	4.0	3.5	5.1
Education			
Student	1.2	0.7	3.5
8/9 yrs of schooling	23.7	24.4	37.0
10 yrs of schooling	32.2	32.6	28.8
12/13 yrs of schooling	42.4	42.1	25.8
No education	0.3	0.2	4.1

1 Federal Statistics Office (December 2009).

### Ethics Statement

The study was approved of by the Ethics committee of Leipzig University (Ethik-Kommission an der Medizinischen Fakultät der Universität Leipzig). Since it was a telephone-based survey, participants were verbally informed on the purpose of the study and then asked for consent to participate. Respondents were informed verbally of the focus of the study and following publications in journals. USUMA, the conducting market research institute, documented the consent and refusal of each participant within the computer-assisted interview. The ethics committee specifically approved this procedure.

### Sample

The overall sample consisted of 3,003 persons. In order to obtain this number, 5,897 civilian individuals were randomly selected. Of these, 32.6% (n = 1,998) refused to participate in the interview. 16.5% of the selected households could not be reached, leading to a response rate of 50.9%. Response rates in this range have been reported before as typical. Previous telephone interview studies showed overall response rates of 55% to 69% [10]; [20]. After weighting, data were representative of the German population concerning age and gender.

### Measures

#### Experimental manipulation by vignettes

Concordantly with commonly used methods in stigma research, experimental manipulation was realized by vignettes. A methodological review recently suggested use of vignettes and following rating scales in order to overcome biased self-report [21]. In previous studies, vignettes have been used to induce vivid pictures of the depicted individuals, especially in the field of mental health research [18]; [22] and attribution theory [23]. Vignettes were derived from previous research, identifying age and gender of obese individuals as potential moderating variables in stigmatization processes [24]. As in previous research, the ages of the vignettes were specified within focus groups [25]. Feedback on proposed ages for a “typical” school child, adult and senior citizen was unanimous [26]. Wording of the vignettes was discussed with experts within the field and members of the USUMA study team.

All six vignettes described an obese individual, varying in gender (female/male) and age (9-year-old child, 42-year-old adult and 68-year-old senior citizen). Weight and height of the vignette were introduced, chosen to be a BMI of 32 kg/m^2^ for the adult and senior vignette, ranging above the 95^th^ percentile of weight for the child vignette, all indicating obesity. This was emphasized by mentioning that the introduced person was “strongly overweight”. In a mixed design, at the end of the interview, a matched vignette regarding age and gender was introduced; however, this time describing a normal-weight person. Each vignette was introduced to an equal number (n = 500) of participants. The vignettes were followed by 2 blocks of vignette-specific questions. The normal-weight vignette was only followed by the scale on stigmatizing attitudes.

#### Stigmatizing attitudes

The short form of the Fat Phobia Scale (FPS) by Bacon et al. (2001) was used to assess stigmatizing attitudes [27]. The short version of the original instrument was derived from factor analysis, representing a factor that describes negative attitudes and showed high correlation with the original long form. It was necessary to use a rating scale of this kind to ensure equal instruments for the different vignettes. It was distributed to all respondents. These rating scales have demonstrated great utility in vignette research [24]. The scale consists of 14 pairs of adjectives on a semantic differential. The interviewer introduced the scale as looking like a ruler with opposing adjectives on each side. The respondent was then asked where on this ruler he/she would rate the vignette on a scale from 1 to 5. Translation of the scale was done following TRAPD (Translation, Review, Adjudication, Pre-Testing and Documentation) guidelines as proposed in social surveys [28]. Pre-Testing was done in qualitative focus groups.

A mean FPS score was calculated, with higher scores indicating higher negative attribution. Participants with more than 5 missing values were excluded. Mean scores of the translated version were comparable to those of the original (M = 3.65, s.d. = 0.49); internal consistency was slightly lower (Cronbach's α = 0.79 compared to α = 0.87 in the original version). Factor analysis supports a one factor solution (Eigenvalue of factor 1 = 3.79).

#### Causal Attribution

Based on previous research and focus groups, 14 items on causes of obesity were presented without further explanation [9]. Within the focus groups, open questions on causes of obesity were asked and participants were asked to identify the most relevant. Items were excluded when the majority of participants found them misleading or not applicable [26]. The interview schedule itself included a further open question to ensure that no information was lost. In the CATI, respondents were asked to rate importance of each potential cause of obesity for the presented vignette on a scale from 1 = “not important at all” to 5 = “highly important”. Factor analysis of all items suggested a three factor solution (Kaiser Criterion of Eigenvalues >1). It was conducted across all age groups in order to identify global underlying structure. Items loading high on Factor 1 can be summarized as causes beyond the individual's control (social environment, cultural influences, advertisement, upbringing and plenteousness of food offers), thus as external causes. Factor 2 includes items directly associated with the individual (quantity of food, willpower, lack of activity behavior) while factor 3 represents genetic and pathogenic influences (genetics, metabolism). A mean score was calculated for each factor.

As an additional proxy, participants were asked to evaluate responsibilities for the solution of the obesity problem (1 = society is responsible to 5 = individual efforts ought to be taken) as done in a previous study [10].

#### Socio-demographic Variables

Socio-demographics were assessed with a standardized questionnaire provided by USUMA. BMI was calculated from self-reported weight and height for the respondent. To avoid missing values on the BMI variable, the CATI data mask calculated a range of weight according to weight classification (normal-weight <24.9 kg/m^2^, overweight 24.9–29.9 kg/m^2^ and obese ≥30 kg/m^2^) by the guidelines of the National Institute of Health [29] when the participant only reported height. Respondents were then asked to pick the range of their actual weight. This procedure led to only six missing values on the categorization variable of weight.

### Data analysis

All analyses were performed using STATA 11 [30]. Wilcoxon sign-ranked test and ANOVA were used to test for significant mean differences. Theory derived potential determinants of stigmatizing attitudes were introduced block-wise to the regression. In all analyses, “no response” codes were treated as missing values. The calculated BMI was categorized according to guidelines (underweight <18.5 kg/m^2^, normal-weight ≥18.5 & <24.9 kg/m^2^, overweight 24.9–29.9 kg/m^2^ and obese ≥30 kg/m^2^). Age was introduced as a continuous variable. Other variables were educational status (no degree, 9^th^ grade degree, 10^th^ grade degree, 12^th^ grade degree), net household income per month (under €999, €1000–1999, €2000–2999, over €3000), residence (former Eastern part of Germany, Western part of Germany), existence of an overweight partner (yes/no), and migrational background (both parents or respondent born in other country, [31]). Variables, representing gender (all male vs. all female vignettes, regardless of age) and age category (children vs. adults vs. senior citizens, regardless of gender) of the vignette, were created.

In previous research, the mean FPS score was categorized. A score of below 2.5 indicates neutral attitudes towards the described person, while a score of 2.5 or higher reflects a higher level of negative attitudes [32]; [33]. For regression analysis, a continuous mean score served as the dependent variable. Obviously, controlling for the evaluation of the normal-weight vignette (FPS score 2) was also necessary to assess systematic rating tendencies regardless of vignette.

In analyses, “no response” or answer refusal categories were treated as missing values. Of 3,003 respondents, 109 had more than 5 missing values on the FPS (either normal- or overweight vignette) and were excluded from analysis, leaving 2,894 individuals for descriptive analyses. For all regression analyses, participants with missing values on independent variables were excluded as well. Multiple regressions were then conducted with 2,459 individuals. All analyses were weighted in order to ensure representativity regarding age and gender.

## Results

### Socio-demographics

Table 1 depicts a comparison of our sample to the German general public. Our sample was slightly older and higher educated. The remaining sample for regression analyses differed only in proportion of women, leading to a better correspondence with the general population. Age and educational status did not differ. Table 2 summarizes BMI characteristics of the total sample. On average, participants were 51.9 years old (s.d. = 18.0, range 18–97 years) and had a mean body mass index (BMI) of 25.6 kg/m^2^ (s.d. = 4.7, range 15–66 kg/m^2^), calculated from self-reported height and weight. 50.7% of all participants were under- or normal-weight, with prevalence of overweight reaching 34% and obesity 15%. Almost half of all male participants (42.4%) were overweight, while 14.8% was obese. For women, the rate of overweight was lower (26.5%), but slightly higher for obesity (15.7%). Obesity was seen as a major health problem by 48.9%.

**Table 2:** Body Mass Index (BMI) categories in the final sample.

Variable	Frequency (%)		
BMI Categorization		Women	Men
Underweight	61 (2.0)	45 (2.9)	16 (1.1)
Normal-weight	1 458 (48.7)	868 (55.0)	590 (41.6)
Overweight	1 020 (34.0)	419 (26.5)	601 (42.4)
Obesity	458 (15.3)	248 (15.7)	210 (14.8)

Under and normal-weight <24.9 kg/m^2^, overweight 24.9–29.9 kg/m^2^ and obese ≥30 kg/m^2^.

### (a) Stigmatizing attitudes and answer distribution

In a first step, stigmatizing attitudes and the distribution of mean FPS scores were to be investigated. The average FPS score of the overweight vignette was 3.65 (s.d. = 0.49, scale range from 1 = positive attribute to 5 = negative attribute) indicating negative attribution overall. The normal-weight vignette yielded an average score of 2.38 (s.d. = 0.46), reflecting neutral attitudes. In Wilcoxon matched-pairs signed-ranks test, the difference in means turns out to be highly significant (z = 45.558, p<0.001), showing the overweight vignette to be rated consistently more negatively. Table 3 shows the means for each adjective, categorized by vignette. Paired t-tests were used to test for significant differences. All adjective comparisons are more negative in the overweight vignette (p<0.001 for each adjective). The five adjective pairs that are rated most negatively are “fast-slow”, “active-inactive”, “strong-weak”, “shapeless-shapely”, and “secure-insecure”.

**Table 3:** Mean for each adjective pair of the Fat Phobia Scale.

	Pair of adjectives	Overweight vignette		Normal-weight vignette		
		Mean	SD	Mean	SD	p
1	Lazy…industrious	3.27	.830	2.40	.836	<0.001
2	No will power… has willpower	3.57	.949	2.31	.887	<0.001
3	Attractive…unattractive	3.61	.958	2.26	.874	<0.001
4	Good self-control…poor self-control	3.48	.935	2.37	.876	<0.001
5	Fast…slow	3.82	.977	2.19	.907	<0.001
6	Having endurance…having no endurance	3.87	.995	2.12	.969	<0.001
7	Active…inactive	3.78	.936	2.00	.914	<0.001
8	Weak…strong	3.33	.986	2.43	.887	<0.001
9	Self-indulgent…self-sacrificing	3.52	.918	2.60	.778	<0.001
10	Dislikes food…likes food	4.11	.935	3.26	.865	<0.001
11	Shapeless…shapely	3.67	1.115	1.99	.905	<0.001
12	Undereats…overeats	4.15	.865	2.83	.552	<0.001
13	Insecure…secure	3.44	.983	2.28	.904	<0.001
14	Low-self-esteem…high self-esteem	3.48	.971	2.29	.842	<0.001

n = 2,875.

Scale as presented in the interview, items 3, 4, 5, 6, 7, 10, and 12, scored as follows:

1 2 3 4 5, items 1, 2, 8, 9, 11, 13, and 14, score as follows: 5 4 3 2 1.

SD – Standard Deviation.

Fat Phobia Scale (FPS) score of the overweight vignette was from 1 = positive attributes to 5 = negative attributes.

When categorized as described in the methods section, table 4 shows the distribution of answers for both types of vignettes, as well as subcategories. We find an exact opposite distribution of answers with the highest numbers in the negative category for the overweight vignettes and in the neutral category for the normal-weight vignettes.

**Table 4:** Answer distribution patterns of mean FPS score.

Vignette	Neutral (FPS ≤2.49)	Negative attributes (FPS≥2.50)	Average FPS score (mean, SD)
	n (%)	n (%)	
Overweight	23 (0.8)	2,871 (99.2)	3.65 (0.49)
Child	3 (0.3)	961 (99.7)	3.75 (0.47)
Adult	12 (1.8)	954 (98.8)	3.62 (0.51)
Senior	8 (0.9)	956 (99.2)	3.60 (0.49)
Normal weight	1,495 (52.0)	1,380 (48.0)	2.38 (0.46)
Child	483 (50.6)	471 (49.4)	2.40 (0.45)
Adult	539 (56.1)	422 (43.9)	2.33 (0.48)
Senior	473 (49.3)	487 (50.7)	2.41 (0.46)

FPS – Fat Phobia Scale, SD – Standard Deviation.

Fat Phobia Scale (FPS) score of the overweight vignette was from 1 = positive attributes to 5 = negative attributes.

### (b) Causal attribution

Secondly, the public's view on causal attribution was assessed. As described in the methods section, three - internal, external and genetic - factors of causal attribution were extracted. Table 5 summarizes mean agreement scores for each extracted factor. Internal factors were rated with highest agreement rates as a cause for the vignette's obesity. Lack of activity behavior and eating too much were the most supported causes. Wilcoxon matched-pairs signed-ranks test was used to test for differences in relevance. While internal factors are seen significantly more relevant (z = −42.155, p<0.001 in comparison to external; z = 37.264, p<0.001 in comparison to genetic factors), external and genetic factors were rated equally important (z = 0.942, p = 0.346). Pearson's correlation was used to show external and internal attribution to be significantly associated (r = 0.35, p<0.001).

**Table 5:** Mean agreement rate for each potential cause of obesity.

Scale	Mean (SD)
**Internal**	
Lack of activity behavior	4.24 (0.78)
Eating too much	4.11 (0.88)
Lack of willpower	3.46 (1.04)
Total Scale	3.94 (0.65)
**External**	
Cultural influences	2.52 (1.02)
Social environment	3.33 (0.99)
Errors in upbringing	3.20 (1.08)
Misleading advertisement and product labeling	3.14 (1.16)
Abundance of food	3.26 (1.12)
Total Scale	3.09 (0.69)
**Genetics**	
Genetic Factors	3.00 (0.98)
Endocrine and metabolic factors	3.13 (1.05)
Total Scale	3.06 (0.87)

SD – Standard Deviation.

Causal Attributes was from 1 = not important at all to 5 = highly important.

#### Causal attribution according to age of vignette

Next, we examined whether different causal attribution according to vignette were present. ANOVA analysis with post-hoc Scheffé tests revealed significant differences: For the vignette of the child, the influence of external and genetic causes was seen differently. Genetic causes were less often agreed on for the child compared to adults and senior citizens (F(2, 2976) = 48.94, p<0.001, Scheffé p<0.001). External causes, however, were seen as more important for the child's obesity (F(2, 2996) = 145.38, p<0.001, Scheffé p<0.001). For internal causation, only the child and the adult vignette differed; ascribing more individual (internal) responsibility to the adult (F(2, 2996) = 3.84, p = 0.022, Scheffé p = 0.023).

#### (c) Determinants of stigmatizing attitudes

In a multivariate analysis, relevant socio-demographic as well as other associations with the different vignettes were investigated. Variables of interest as described above were successively introduced into the regression model and are shown in table 6. Model 4 is the full model, retaining a total of 22 variables and accounting for a total of 28% of the variance. Only significantly associated variables are shown. Of socio-demographic influences, only higher education (all categories referred to no educational degree) showed a significant association with a more positive view of the overweight vignette. Compared to normal-weight participants, overweight and obese individuals as well as participants with an obese or overweight partner showed lower negative attribution scores. A higher (e.g. negative) score in the attribution of the normal-weight vignette (FPS score 2) was associated with lower average scores for the overweight vignette.

**Table 6:** Prediction of stigmatizing attitudes.

	Model 1	Model 2	Model 3	Model 4
Education (ref = no degree)				
Secondary General School (9th grade)	−0.356*** (0.0994)	−0.287** (0.0917)	−0.246** (0.0907)	−0.278** (0.0852)
Secondary Intermediate School (10th grade)	−0.319** (0.0978)	−0.244** (0.0902)	−0.214* (0.0892)	−0.251** (0.0838)
Upper Secondary School	−0.311** (0.0974)	−0.184* (0.0900)	−0.171 (0.0889)	−0.214* (0.0835)
FPS score 2 (normal-weight vignette)		−0.421*** (0.0202)	−0.414*** (0.0200)	−0.374*** (0.0191)
Personal body weight (ref = normal-weight)				
Underweight			−0.0365 (0.0659)	0.00859 (0.0620)
Overweight			−0.0752*** (0.0211)	−0.0680*** (0.0198)
Obesity			−0.202*** (0.0267)	−0.186*** (0.0252)
Overweight Partner (no/yes)			−0.0649* (0.0323)	−0.0597* (0.0304)
Age of vignette (ref = child)				
Adult				−0.144*** (0.0236)
Senior Citizen				−0.104*** (0.0238)
Perceived cause of obesity (mean values)				
External				0.0721** (0.0277)
Internal				0.126*** (0.0263)
Genetics				−0.0195 (0.0171)
Interaction age of vignette and perceived causes				
Adult * internal causes				0.135*** (0.0362)
Senior * internal causes				0.0757* (0.0359)
Constant	3.928***	4.904***	4.912***	4.946***
	(0.103)	(0.106)	(0.105)	(0.104)
Observations	2459	2459	2459	2459
Adjusted *R* ^2^	0.005	0.155	0.176	0.276

FPS—Fat Phobia Scale; Standard errors in parentheses;

Additional adjustments (all insignificant predictors):

Model 1: gender, age, income, residence, migrational background.

Model 2: same as model 1.

Model 3: gender of the vignette and perceived responsibility of problem solution, interaction effects causes (external/genetics) * age of vignette.

* *p*<0.05,

** *p*<0.01,

*** *p*<0.001.

Attribution to internal as well as external factors proved to be associated with higher stigmatizing attitudes. As indicated descriptively (Table 4), the vignette of the obese child was rated far more negatively compared to that of an adult or senior citizen (p<0.001). Looking into causes ascribed to obesity, we found that an interaction effect of the age of the vignette and internal causal attribution exists. The higher the age of the vignette is, the higher the influence of internal causes on the negativity of ratings.

## Discussion

The aim of this study was to determine prevalence of stigmatizing attitudes in the general population towards obese individuals and the analysis of associated factors.

### Socio-demographics

Our sample was representative of the German population with respect to age and gender. Especially regarding prevalence of obesity, our findings compare to those of previous studies. The most recent study by the Federal Ministry of Food, Agriculture and Consumer Protection and Federal Research Institute of Nutrition and Food found 20.5% of all men and 21.7% of all women to be obese [34]. Using self-report as a measure created a probable underestimation of about 6% in overall prevalence. Previous research has shown that commonly, self-report height is over-reported while there is an under-reporting of self-report weight. In total BMI, it is calculated −0.4 to −1.0 kg/m^2^ lower than the actual BMI of each individual [35]. The ratio of men to women in prevalence rates, however, was reproduced: obesity was reported by 14.8% of all men and 15.7% of all women in our sample. Also, a response bias might a possible reason for the lower prevalence rates. It might be that obese individuals are less likely to participate in a survey on health matters. Furthermore, our sample showed a link of a higher educated and higher socio-economic status to lower prevalence of obesity [36].

### (a) Stigmatizing attitudes and answer distribution

The attribution of negative adjectives to obese individuals as an indicator for stigmatizing attitudes is highly common in the German public. The obese vignettes were rated significantly more negative by study participants. Obese individuals are rated negatively by 99% of the population. Although it is difficult to compare our findings to previous studies, distribution analysis of stigmatizing attitudes done in the past showed more of a normalized distribution (equal amounts of non-stigmatizing attitudes, neutral and stigmatizing attitudes) [10]. This might be an effect of the more indirect way to assess negative attitudes by the method used (semantic differential). Hilbert et al. used a subscale of the Antifat Attitudes Test, where rather blunt phrases (such as “Fat people have no willpower”) have to be rated [37]. In the present study we chose a vignette-driven approach, assuming the influence of social desirability in answers to be lower.

The German version of the Fat Phobia Scale showed excellent psychometric properties and could be an adequate measure to determine stigmatizing attitudes in future research. The average score corresponded to those found in previous samples [27]. The significant difference in vignette-ratings indicated that the vignettes differing only in BMI worked as an inducement of a picture. The significant association of lower FPS score of the obese vignette by the FPS score of the normal-weight vignette indicates a tendency of rating both vignettes equally in form of systematic bias. Respondents there seem to rate the two vignettes equally to the natural mid-point of the scale.

When looking into individual adjectives that are ascribed to obese individuals, insecurity is strongly seen as a feature. Likewise, activity-related adjectives are supported for the overweight vignette such as “fast-slow” and “active-inactive”. Quite impressively, there is not a single adjective pair that is rated equally over the two vignettes. Internal, character-based adjectives, such as having no self-control and being self-indulgent, are seen as fitting for the obese vignettes more often as well.

### (b) Causal attribution

Two internally rated items (overeating, lack of activity) are endorsed more often compared to genetic influences. This goes in line with previous research that found obesity to be seen as a controllable condition [38]. The importance of obesity-influencing factors differed by vignette.

#### Causal attribution according to age of vignette

For the children vignettes, less individual responsibility was ascribed, representing the fact that children are much more likely to be influenced by parenting style and social surroundings. They are obviously seen as not being able to control their condition. Especially for the adult vignette, individual responsibility is rated as highly important. We did, however, find internal and external factors to be inter-correlated. Genetics and diseases as independent influencing factors seem to be reliably extractable; however, external and internal influences are not as easily differentiated. This seems especially true, since the external influences scale used only consisted of items that apparently are out of the individual's control (advertisement, upbringing, cultural influences) but might not be viewed as such by the general public.

### (c) Determinants of stigmatizing attitudes

As results from studies in representative samples on the stigma of obesity and its determinants are mainly lacking [9], integration in previous findings is limited. Contrary to previous findings, age was not significantly associated with stigmatizing attitudes. Comparability is limited, though. In a study where such an association was found, Rand et al. (2000), experimental line-drawing was used, while in a representative study, age was not used in a multiple regression analysis [10]; [39]. The same is true for the other significant socio-demographic association, educational attainment. Higher education led to lower FPS scores in our sample as well, but categorizing into four categories might not differentiate enough between different stages of educational attainment, namely because 9^th^ and 10^th^ grade degrees are quite comparable in Germany. Participants with lower educational attainment showed higher rates of overweight and obesity; personal weight therefore might account for the effect found. Being overweight or obese is associated with lower scores on the FPS. This contradicts assumptions that self-stigma in mental illness is conferrable to obesity. Self-stigmatization describes the phenomenon that stigmatized individuals internalize negative attributes ascribed to them, regarding them as fitting. For that reason, self-stigmatization is more commonly referred to as internalized stigma [40]. Results might be an indicator that internalized stigma does not play a significant role in obese individuals. However, this finding needs further research.

Likewise, the familiarity with the condition based on one's own overweight and obesity might explain this finding. In psychiatric research, it has been shown that contact to a person with a psychiatric disorder does indeed reduce social distance and stigma [41]; [42].

#### Causal attribution

Higher agreement rates in both external and internal attribution of obesity causes predict higher stigmatizing attitudes. Hilbert et al. (2008) found the same pattern and concluded that this is not a result contrary to attribution theory, since environmental factors are obviously linked to the individual [10]. Situational factors as such do contribute to individual choices and development and may not be viewed as entirely out of the individual's control.

#### Associations to vignettes

The inter-correlation of the construct of causal attribution might also be the determining factor in another unexpected result. Contrary to attribution theory assumptions – expecting children to have no fault for their overweight and obesity and thus resulting in lower stigmatizing attitudes – results show the vignette of a child to be the subject of higher negative attributions compared to the middle- and old-age vignettes. Even under control of causal attribution and an interaction effect that shows that the influence of internal causes increases with the vignette's age, obese children are seen far more negatively. The effect does not vanish in the model, and it was not possible to find an explanatory variable.

#### Child vignette

Weight stigma transported by adults towards obese children has not been investigated yet. A comprehensive review by Puhl and Latner (2007) summarizes data on peer relationships as well as educators' and parents' views of obese individuals, but only few studies have investigated adult views on obese children [16]; [43]; [44]. Contrary to these previous findings, we found obese children to be rated less favorable by adults. An association of blaming children for their condition and higher rates of dislike were described equally, however [16]. Adams and colleagues (1988) were able to show a more negative view on the picture of an obese child compared to a picture of a normal-weight child by adults [45]. Obese children were likewise the least preferred in another study [44]. Our findings reproduce that result on a much larger representative database, but, furthermore, indicate that obese children are subject to more negative attribution compared to adults and senior citizens beyond explanation approaches such as the attribution theory. One aspect might be the altered view of the public on the importance of obesity prevalence. Public awareness has changed substantially. During the early 2000s only 2 to 3 per cent of the population considered obesity to be one of the most important health issues [46], while nowadays in our sample almost half of the respondents agree strongly on this issue. Likewise, the elevated risk for co-morbid diseases in obesity is strongly emphasized by an equal amount of participants. This said, it might be that the possibly devastating future effects of obesity, especially in young children, are seen by the public, and the social aspect of stigmatization gains importance. Stigma might play a normative role, serving to enforce compliance with existing social norms to push individuals to adjust their behavior to those standards [47], and the public possibly sees the need to change that behavior first and foremost within the children.

This result is especially important when considering consequences of stigmatization, among them psychological disorders, unhealthy eating and activity behavior and stress-induced pathophysiology [7]. These effects have mainly been shown in stigmatized adult populations, but can be expected in children as well [43].

### Limitations

This study is limited by a relatively low response rate, which, however, is common in telephone research. The response rate corresponds to that found in a previous representative sample [10]. Numbers were further reduced substantially due to missing data, but respondents still reflected the composition of the German general public. Bodyweight and –height were assessed via self-report. Prevalence rates of overweight and obesity correspond to previously reported estimates. Since prevalence rates have risen since the 2005–2007 survey, and in light of the influence of self-report [1]; [34]; [48], we may deal with an underestimation of rates. This seems true for the absolute prevalence rates of obesity, while the prevalence rate difference between men and women was found similar, rates differed by about 6 per cent. Furthermore, this study was not able to investigate appearance-related stigma, since we only used verbal vignettes. We were not able to measure actual stigmatizing behavior, but had to rely on questionnaire-based responses. Aside from the vignettes used, it would have been interesting to examine effects on vignettes within their teenage years and in young adulthood. Since studies on these are still lacking, they ought to be focus of forthcoming projects.

### Conclusions

Findings address the usefulness of this study in planning public health campaigns to prevent stigmatization. It seems that anti-stigma interventions will need to aim at obese children just as much or even more than focusing on the obese adult. In light of the enormous consequences of perceived stigmatization and discrimination, the need for targeted anti-stigma interventions is unquestionable. Obviously, the implementation of an adequate etiological model might still be a base for anti-stigma intervention. Public awareness of the complexity of the causes of obesity and the difficulties of affected individuals in achieving sustainable weight loss needs to be raised in order to reduce stigmatization. Yet, other approaches, considering the flaws of attribution theory, need to be taken into consideration. Changing beliefs on the controllability of the condition in intervention has only yielded limited success [49], making other paths such as changing the normative aspects of obesity the object of consideration. Results show that obesity is still an undesired condition within society, associated with negative attributes. Social consensus theoretical approaches, making obesity a more accepted condition, might be a starting point for more effective interventions [50]. Furthermore, according to the theory of planned behavior, social norm of preference of normal-weight individuals might hinder obese individuals to seek help for their condition. Especially anticipated stigmatization by health care professionals seems to be a barrier to help-seeking [51], making this another very relevant area for further research.
